# Geographic Distribution of Human Infections with Zoonotic *Ancylostoma ceylanicum* and Anthropophilic Hookworms in Ecuador: A Retrospective Analysis of Archived Stool Samples

**DOI:** 10.4269/ajtmh.23-0469

**Published:** 2024-01-23

**Authors:** Dayana Aguilar-Rodríguez, Victor Seco-Hidalgo, Andrea Lopez, Natalia Romero-Sandoval, Manuel Calvopiña, Angel Guevara, Lucy Baldeón, Alejandro Rodríguez, Rojelio Mejia, Thomas B. Nutman, William J. Sears, Philip J. Cooper

**Affiliations:** ^1^ School of Medicine, Universidad Internacional del Ecuador, Quito, Ecuador;; ^2^ Institute of Infection and Immunity, St. George’s University of London, United Kingdom;; ^3^ Grups de Recerca d’Amèrica i Àfrica Llatines – GRAAL, Barcelona, Spain and Quito, Ecuador;; ^4^ One Health Research Group, Faculty of Medicine, Universidad de las Americas, Quito, Ecuador;; ^5^ Instituto de Biomedicina, Universidad Central, Quito, Ecuador;; ^6^ National School of Tropical Medicine, Baylor College of Medicine, Houston, Texas;; ^7^ Laboratory of Parasitic Diseases, National Institutes of Health, Bethesda, Maryland

## Abstract

Zoonotic human infections with *Ancylostoma ceylanicum* have recently been reported in the Americas. We used archived human stool samples to study the geographic distribution of human infections with *A. ceylanicum* and anthropophilic hookworms in different geoclimatic regions (coastal, Andean, and Amazon) of Ecuador. We analyzed retrospectively archived human stool samples from five studies previously screened for hookworm infection by microscopy, of which four included hookworm-positive samples only and one involved hookworm-negative samples to increase geographic distribution of sampling. Stools were analyzed using multi-parallel quantitative polymerase chain reaction (qPCR) assays to detect *Necator americanus*, *Ancylostoma duodenale*, *A. ceylanicum, Ascaris lumbricoides*, *Trichuris trichiura*, and *Strongyloides stercoralis*. Sequencing was done for the *A. ceylanicum cox1* gene. A total of 132 samples were analyzed, of which 69 (52.3%) were from hookworm-positive and 63 (47.7%) from hookworm-negative individuals by microscopy. Overall, 82.6% of microscopy-positive samples and 33.3% of microscopy-negative samples were positive for hookworm by qPCR. Of microscopy-positive samples, 36.2% were *A. ceylanicum*, 37.7% *A. duodenale*, and 33.3% *N. americanus*, whereas equivalent proportions for microscopy-negative samples were 1.6%, 31.7%, and 1.6%, respectively. *Ancylostoma duodenale* was the most widely dispersed geographically, followed by *N. americanus*. *Ancylostoma ceylanicum* was least dispersed but was detected in coastal and Amazon regions. In conclusion, human infections with *A. ceylanicum, A. duodenale,* and *N. americanus* were detected in different geoclimatic regions of Ecuador. Additional studies are required to further define the epidemiology of human *A. ceylanicum* infections, but the potentially widespread presence of this helminth in human populations in Ecuador has implications for hookworm control strategies.

## INTRODUCTION

Human hookworms are soil-transmitted helminths (STHs) estimated to infect 229 million people with a worldwide distribution.^1^ Hookworms are most prevalent in tropical regions of low- and middle-income countries in the poorest communities without access to clean water and sanitation.^2^ Hookworms feed on blood in the mucosa of the small intestine and are a leading cause of chronic anemia affecting primarily young children and women of childbearing age. Hookworm infections are estimated to cause the loss of 845,000 years lived with disability annually.^1^ Humans can be infected with several species of hookworms, including *Ancylostoma duodenale* and *Necator americanus*, and with zoonotic hookworms, principally *Ancylostoma ceylanicum*.

*Ancylostoma ceylanicum*, although a natural infection of dogs and cats, is now recognized as the second-most frequent hookworm species infecting humans after *N. americanus* and is estimated to infect 100 million humans primarily in Asia.^3^ Although *A. ceylanicum* can be distinguished morphologically from other hookworm species, our understanding of its epidemiology in animal and human populations has required the application of modern molecular methods.^3^ Autochthonous infections with *A. ceylanicum* in the Americas have only recently been described in humans in Ecuador^4^ and dogs in Grenada in the Caribbean.^5^

Human infections with *A. ceylanicum* are thought to follow a clinical course similar to those of the anthroponotic hookworms characterized by “ground itch” at the site of percutaneous larval entry, abdominal symptoms, and eosinophilia.^6^ Acute symptoms may be more severe with *A. ceylanicum* than for the other hookworms,^6^ and chronic high-intensity *A. ceylanicum* infections can cause anemia.^3^ Although greater morbidity has been attributed to *Ancylostoma* spp., evidence for differences in risk of anemia attributable to different hookworm species is limited.^2^

Here, we describe the distributions of infections with *A. ceylanicum*, *A. duodenale*, and *N. americanus* in different geoclimatic regions of Ecuador using archived samples from previous studies. Our data demonstrate the presence of human infections with *A. ceylanicum* and the two anthropophilic species in populations from different geoclimatic regions of the country.

## MATERIALS AND METHODS

### Ethics statement.

Informed written consent was obtained from adult participants and from the parent or legal representative of a child. The study protocols were approved by Hospital Pedro Vicente Maldonado, Universidad San Francisco de Quito, and Universidad Central del Ecuador (approvals 13/06/2005, 6/11/2010, 22/11/2013; 26/06/2015; LEC IORG 0001932; IRB 2483 COBI-AMPHI-0064-11; and 038-CE-UCE-2019).

### Study setting, populations, and sampling.

Ecuador lies on the equator and is crossed by the Andes, dividing the country into three distinct geoclimatic regions: western Pacific coastal with subtropical and tropical lowlands, central Andean with high mountains and deep valleys where climates may be temperate to subtropical, and eastern Amazon lowlands of humid tropical rain forest. A total of 132 archived stool samples, stored in 70–90% ethanol, were analyzed from five studies; the first four studies were of microscopy-positive samples for hookworm, whereas the fifth study was of microscopy-negative samples for hookworm.

The first four studies included 69 hookworm-positive stool samples (criteria for inclusion of samples in this analysis were being microscopy positive [or quantitative polymerase chain reaction (qPCR) positive for hookworm in the cohort subsample described below] and having a suitably stored sample available for analysis). Study 1 involved 57 samples from a birth cohort (ECUAVIDA cohort) of 2,404 mestizo and Afro-Ecuadorian children who were followed up to 8 years of age in the rural district of Quinindé in Esmeraldas Province (northern coastal). This population-based cohort was designed to study the effects of early life infections on the development of allergy and allergic diseases in childhood and is described in detail elsewhere.^7^ Newborns were recruited at the public hospital serving the district between 2006 and 2009, and children were followed up to their eighth birthday. A total of 13,354 stool samples were collected longitudinally at 3, 13, 18, 24, 30, 36, 60, and 96 months of age between 2005 and 2017^8^ and examined by a combination of microscopic methods (direct, Kato-Katz, formol-ether concentration, and carbon coproculture). Stool samples collected between 7 months and 8 years from a subsample of cohort children (selected from a random sample of 400 children with stool samples collected at 13 months of age) were examined by multi-parallel qPCR for STHs including hookworm (*Ancylostoma* spp. and *N. americanus*) as described in detail elsewhere.^9^ The results of these previous polymerase chain reaction (PCR) analyses of this random subsample were used to select samples for inclusion in the present analysis in combination with the findings of stool microscopy from the whole cohort. Therefore, 57 samples analyzed from this cohort were those found to be either microscopy positive for hookworm infection and/or positive by PCR (for *Ancylostoma* spp. and/or *N. americanus* in the random subsample) and for which an archived sample (stored in 90% ethanol at −30°C) was available.^8^

Study 2 comprised three hookworm-positive samples from a survey of stool samples collected in 2015 from 58 indigenous Chachi (mean age, 22 years; range, 1 to 65 years) living in a riverine community in the district of Eloy Alfaro in Esmeraldas Province (northern coastal) in which stool samples were examined using a combination of microscopic methods (direct, Kato-Katz, sedimentation, and formol-ether concentration).^10^ Study 3 involved seven hookworm-positive samples from a survey of stool samples collected in 2015 from 192 indigenous Shuar (mean age, 23 years; interquartile range, 2 to 78 years) living in two riverine communities in the district of Tiwintza in Morona-Santiago Province (southern Amazon) in which stool samples were examined using direct, Kato-Katz, and formol-ether concentration methods.^11^ Study 4 included two hookworm-positive samples from a survey of stool samples collected in 2017 from 32 mestizos (mean age, 32 years; range, 1 to 83 years) living in a riverine community in the district of Jipijapa in Manabi Province (central coastal) in which stool samples were examined using the formol-ether concentration method.^12^

To increase the geographic range of sampling within Ecuador, we analyzed 63 hookworm-negative (by microscopy using the Richie method) stool samples, archived in 70% ethanol, from a study done in the coastal and Andean regions in 2017.^13^ The only inclusion criterion for this study was having a suitably preserved and stored sample available that included 1) three samples from indigenous Awa and Afro-Ecuadorian children and adults from riverine communities in the district of Eloy Alfaro in Esmeraldas Province (northern coastal); 2) 16 samples from urban schoolchildren in the district of Balzar in Guayas Province (southern Coastal); 3) 11 samples from urban schoolchildren in Guayllabamba (district of Quito) and Uyumbicho (district of Mejia) in Pichincha Province (northern Andean); and 4) 33 samples from schoolchildren in the city of Cuenca in Azuay Province (southern Andean). In this last study, only an aliquot from a proportion of samples had been suitably archived (i.e., preserved with 70% ethanol); the remaining samples had been stored in 10% formol and were unsuitable for molecular analysis.

### Sample preparation and molecular analyses.

Approximately 50 mg of stool was transferred into lysing matrix tubes. Samples were homogenized using FastPrep-24 (MP Biochemicals, Solon, OH), and DNA was extracted using FastDNA for Soil Kit (MP Biochemicals) according to the manufacturer’s instructions. All samples were extracted after the addition of an internal control as reported previously.^14^ Procedures for PCR detection of STH parasites and *A. ceylanicum* are described in detail elsewhere.^4^ Briefly, high-throughput multi-parallel qPCR reactions were prepared using a ratio of 3.5 μL of TaqMan Fast Advanced Master Mix (Applied Biosystems, Thermo Fisher Scientific, Carlsbad, CA), 2 μL of template DNA, 0.5 μL of water, and 1 μL of a PrimeTime qPCR Probe Assay (Integrated DNA Technologies, Leuven, Belgium) with forward primer, reverse primer, and probe at final reaction concentrations of 500 nM, 500 nM, and 250 nM, respectively (Supplemental Table 1). MicroAmp optical plates (96-well; Applied Biosystems) were used to prepare and run qPCRs for each respective target: *Ascaris lumbricoides/suum*, *A. ceylanicum*, *Strongyloides stercoralis*, *Trichuris trichiura*, *N. americanus*, and *A. duodenale*.^4^^,^^15^^–^^17^ Sample reactions were amplified on a 7500 Fast Real-Time PCR System (Applied Biosystems) with an initial incubation at 95°C for 20 seconds followed by 40 cycles of denaturation at 95°C for 3 seconds and annealing/extension at 60°C for 30 seconds.

### *Ancylostoma ceylanicum* molecular characterization.

A 377-bp region of the *A. ceylanicum* cytochrome c oxidase subunit I (*cox1*) partial gene was amplified^18^ from positive samples detected by qPCR. Polymerase chain reaction was performed in a 15-µL reaction mixture containing 7.5 µL of Platinum^™^ SuperFi^™^ PCR Master Mix (Invitrogen, Carlsbad, CA), 0.4 µM of each primer (Supplemental Table 2), 4.3 µL of water, and 2 μL of template DNA. The PCR conditions used were initial denaturation at 98°C for 30 seconds, followed by 40 cycles of denaturation at 98°C for 7 seconds, annealing at 58°C for 10 seconds, elongation at 72°C for 30 seconds, and a final extension at 72°C for 5 minutes on a MultiGene^™^ OptiMax Thermal Cycler (Labnet International, Edison, NJ). The 377-bp products of PCR were visualized by 0.8% agarose gel. Positive amplicons were sent for Sanger sequencing external service (Macrogen, Seoul, South Korea). The consensus sequence (GenBank accession number ON773157.1) was aligned with those of the samples, and a phylogenetic tree was assembled using the maximum-likelihood method performed on MegaX Software with bootstrapping (×1,000). *Ancylostoma duodenale and Ancylostoma braziliense* sequences (GenBank accession numbers MK271367 and DQ438069, respectively) were used as outgroups.

## RESULTS

### Hookworm and STH infection rates and geographic distributions of hookworm species.

**Figure 1.**
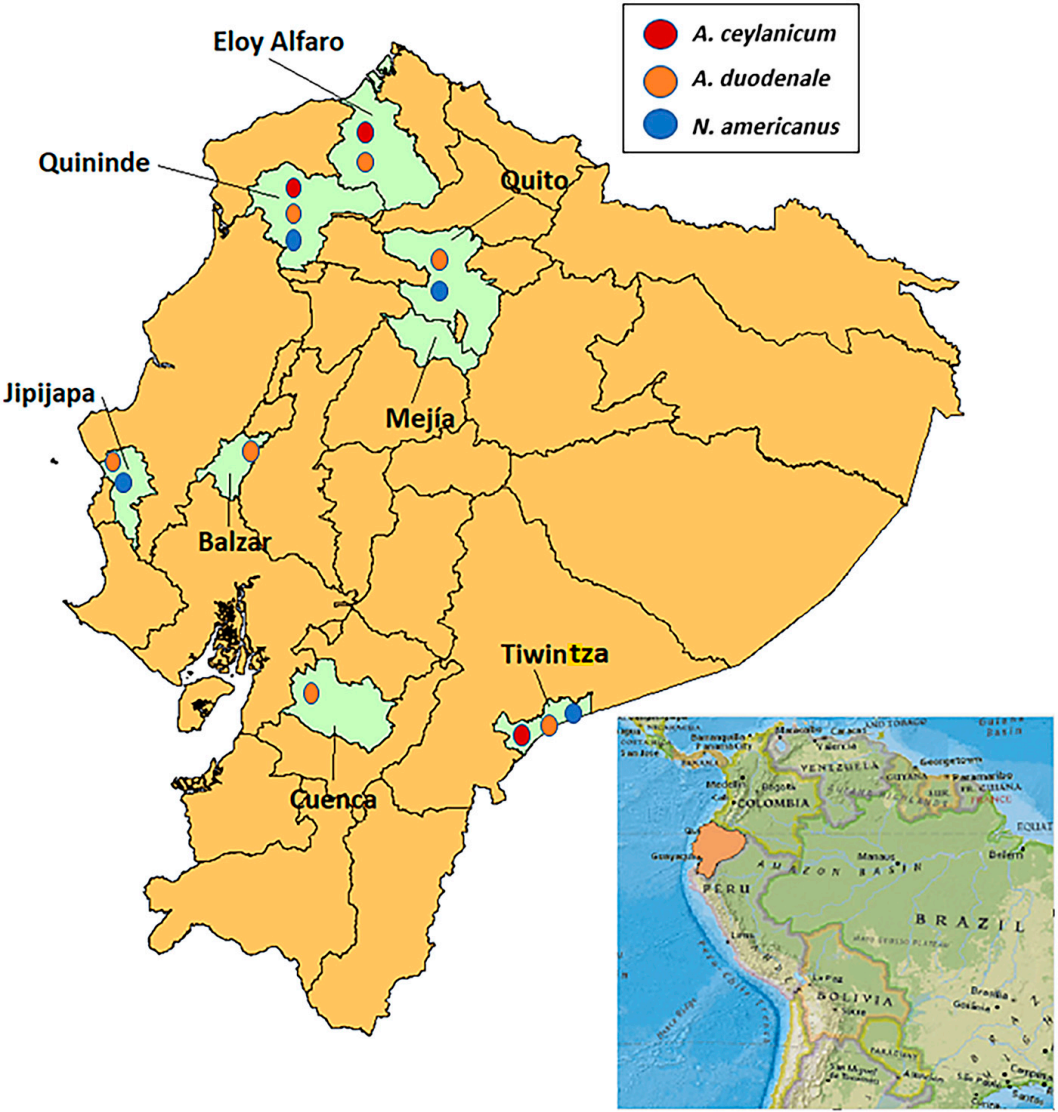
Geographic locations of study sites in Ecuador and presence of the three hookworm species. Samples were analyzed from five studies in seven districts (light green shading) of six provinces. Presence of the three hookworm species (*Ancylostoma ceylanicum* [red circles], *Ancylostoma duodenale* [orange circles], and *Necator americanus* [blue circles]) in the seven districts is shown.

Characteristics of the five included studies and samples used for analysis are shown in Table 1. Table 2 shows results for hookworm-positive and -negative (by microscopy) samples by study using multi-parallel qPCR for detection of *Ancylostoma* spp., *N. americanus*, and other STHs. Of the 69 analyzed samples that were positive for the presence of hookworm eggs by microscopy, qPCR detection rates for the different parasites were as follows: any hookworm (82.6%); two or more hookworm species (23.2%); *A. ceylanicum* (36.2%), *A. duodenale* (37.7%), *N. americanus* (33.3%), *S. stercoralis* (7.3%), *T. trichiura* (30.4%), *A. lumbricoides* (85.5%), and any STH co-infection (95.7%). Of the 63 samples that were negative for the presence of hookworm eggs by microscopy, a significant proportion were positive for the presence of *A. duodenale* (31.7%) by qPCR, although proportions infected with the other hookworms were much lower (1.6% for *A. ceylanicum* and *N. americanus*). Quantitative PCR detection rates for other STH parasites were relatively high except for *T. trichiura* (0%): *A. lumbricoides* (55.6%), *S. stercoralis* (6.3%), and any STH co-infection (66.7%). Overall, *A. ceylanicum* was detected in 19.7% of all 132 stool samples tested from the five studies and was present in three districts of two provinces in coastal and Amazon regions of the country (Figure 1). *Ancylostoma duodenale* was the most frequent hookworm detected overall (34.9% of all samples positive), being present in seven districts of six provinces in all three geoclimatic regions of the country. The proportion infected with *N. americanus* (18.2%) was similar to that for *A. ceylanicum*, but infections appeared to be more geographically spread, being detected in four districts of four provinces in all three geoclimatic regions.

**Table 1:** Characteristics of populations from the five studies for which archived stool samples were used for molecular analyses

Study	District/province	Study design/year	Geoclimatic zone	Ethnicity	Samples (*n*)	Population mean age (range)	Hookworm prevalence in study population (*n*)*
Hookworm positive by microscopy (*N* = 69)							
1	Quinindé/Esmeraldas	Birth cohort/2006–2017	Northern coastal (urban and rural)	Mestizo/Afro- Ecuadorian	57	3 months to 8 years†	0.3% at 8 years (5/1,764 at 8 years)^8^
2	Eloy Alfaro/Esmeraldas	Cross-sectional/2015	Northern coastal (rural)	Indigenous	3	22 (1–65) years	2.9% (3/105)^10^
3	Tiwintza/Morona-Santiago	Cross-sectional/2015	Southern Amazon (rural)	Indigenous	7	23 (1–80) years	3.6% (7/192)^11^
4	Jipijapa/Manabi	Cross-sectional 2017	Central coastal (rural)	Mestizo	2	32 (1–83) years	1.1% (2/176)^12^
Hookworm negative by microscopy (*N* = 63)							
5	Eloy Alfaro/Esmeraldas	Cross-sectional/2017	Northern coastal (rural)	Afro-Ecuadorian	3	Children and adults	0% (0/184)^13^
	Balzar/Guayas		Southern coastal (urban)	Mestizo	16	10 (6–15) years	0% (0/135)^13^
	Quito/Pichincha		Northern Andean (urban)	Mestizo	6	7 (6–7) years	0% (0/76)^13^
	Mejia/Pichincha		Northern Andean (urban)	Mestizo	5	6 (6–8) years	0% (0/92)^13^
	Cuenca/Azuay		Southern Andean (urban)	Mestizo	33	7 (6–8) years	0% (0/97)^13^

* Numbers in parentheses represent total sample size of each survey analyzed using microscopic methods.

† Samples collected from a birth cohort followed up to 8 years with periodic stool collections.

**Table 2:** Results of multi-parallel quantitative PCR for soil-transmitted helminths and hookworm species in stool samples from five studies analyzed with and without hookworm infection detected by microscopic methods

Study (district/province)	Samples (*n*)	Results of quantitative PCR for STH parasites							
		*Ancylostoma ceylanicum*	*Ancylostoma duodenale*	*Necator* *americanus*	*Ascaris lumbricoides*	*Trichuris trichiura*	*Strongyloides stercoralis*	Any hookworm	Any STH co-infection
Hookworm positive by microscopy (*N* = 69)									
1. (Quinindé/Esmeraldas)	57	42.1%	38.6%	26.3%	84.2%	24.6%	7.0%	84.2%	96.5%
2. (Eloy Alfaro/Esmeraldas)	3	0%	33.3%	0%	66.7%	0%	0%	33.3%	66.7%
3. (Tiwintza/Morona-Santiago)	7	14.3%	28.6%	85.7%	100%	71.4%	14.3%	85.7%	100%
4. (Jipijapa/Manabi)	2	0%	50%	100%	100%	100%	0%	100%	100%
Total	69	36.2%	37.7%	33.3%	85.5%	30.4%	7.2%	82.6%	95.7%
Hookworm negative by microscopy (*N* = 63)									
5. (Eloy Alfaro/Esmeraldas)	3	33.3%	33.3%	0%	100%	0%	0%	33.3%	100%
5. (Balzar/Guayas)	16	0%	37.5%	0%	87.5%	0%	25.0%	37.5%	93.8%
5. (Quito/Pichincha)	6	0%	16.7%	16.7%	33.3%	0%	0%	33.3%	33.3%
5. (Mejia/Pichincha)	5	0%	0%	0%	20%	0%	0%	0%	20.0%
5. (Cuenca/Azuay)	33	0%	36.4%	0%	45.5%	0%	0%	36.4%	63.6%
Total	63	1.6%	31.7%	1.6%	55.6%	0%	6.3%	33.3%	66.7%
All samples (*N* = 132)									
5. Five studies	132	19.7%	34.9%	18.2%	71.2%	15.9%	6.8%	59.1%	81.8%

PCR = polymerase chain reaction; STH = soil-transmitted helminth.

Sociodemographic characteristics and STH co-infections among the *26 A. ceylanicum–*positive samples are shown in Supplemental Table 3: median age was 5 years (range, 1 to 15 years), 81% were male, 73% were of mestizo ethnicity (23% Afro-Ecuadorian and 4% indigenous), and 54% had rural residence. Almost all (92.3%) had co-infections with at least one other STH species. Median cycle threshold (Ct) value by qPCR for *26 A. ceylanicum*–positive samples was 21.8 (range, 18.2 to 33.9); for *46 A. duodenale*–positive samples, the Ct value was 15.6 (range, 5.6 to 37.9), and for *24 N. americanus*–positive samples, it was 21.5 (range, 14.2 to 37.6).

### *Ancylostoma ceylanicum* sequence variation.

**Figure 2.**
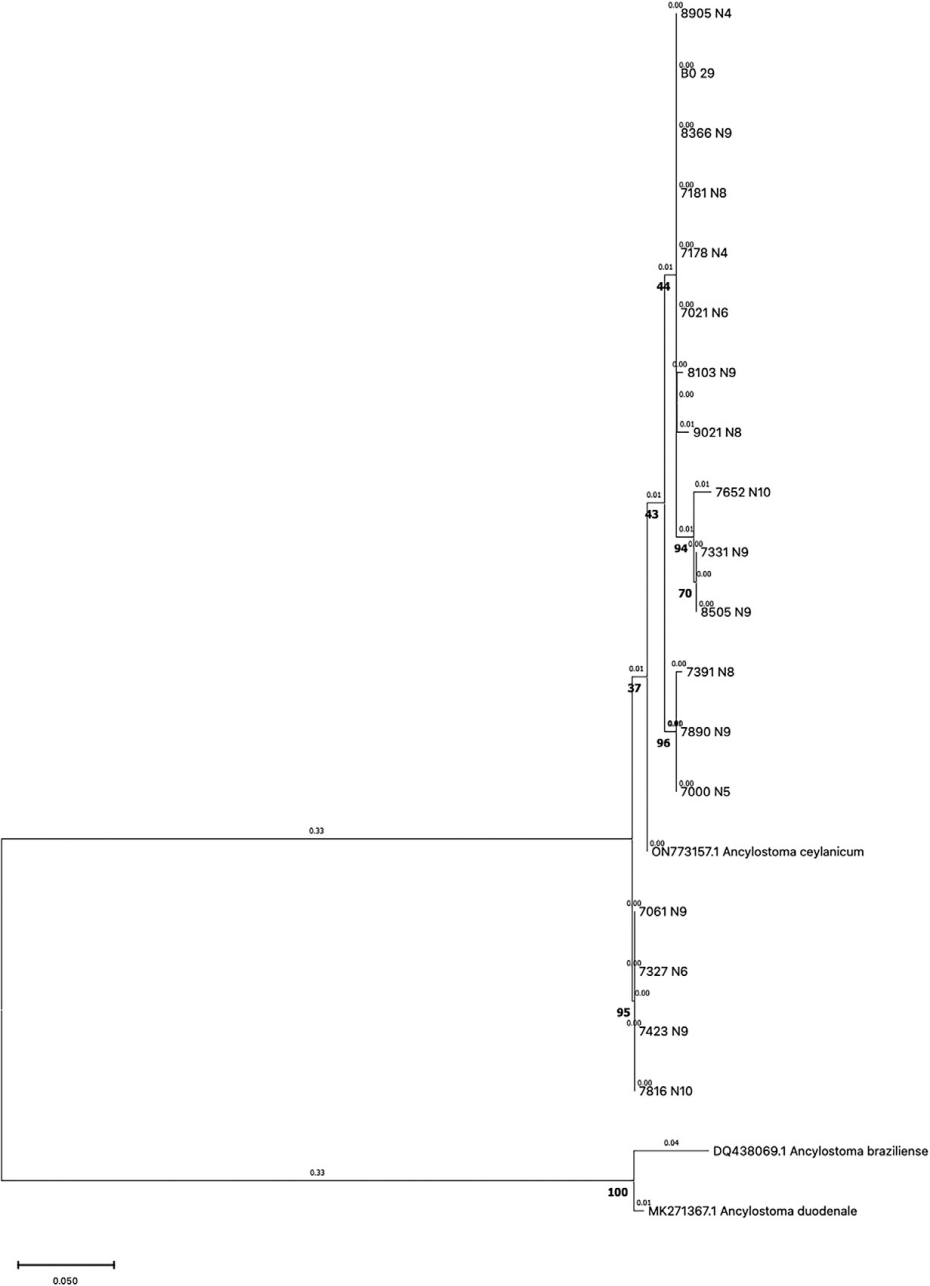
Phylogenetic tree comparing *Cox1* sequences of *Ancylostoma ceylanicum* from 18 subjects with reference sequences. ON773157 was used as the reference sequence from a previous analysis of two samples from infected children in Esmeraldas Province, Ecuador.^4^ *Ancylostoma braziliense* (DQ438069) and *Ancylostoma duodenale* (MK271367) sequences were used as outgroups. Bootstrap values are presented in bold under branches, whereas the ﻿ computed branch lengths are presented in grey above branches. A tree scale is shown (bottom left).

Sanger sequencing was done on positive samples amplifying for the *A. ceylanicum cox1* gene by PCR. Consensus sequences were obtained for 18 samples and were all greater than 98% identical to the corresponding *A. ceylanicum* reference sequence (Genbank accession number ON773157.1) targeted by PCR. Figure 2 shows a phylogenetic tree comparing known Cox1 sequences of *A. ceylanicum*. A distance matrix is provided in Supplemental Table 4. Seventeen of the 18 sequences were from the birth cohort.

### Hookworm infections in the birth cohort.

To explore the epidemiology of zoonotic and anthropophilic hookworms in one of the study settings, we used data from the birth cohort to examine the acquisition of hookworm infections during early childhood using the results of stool microscopy in the whole cohort and qPCR in the random subsample. Data were available from 2,398 children (99.8%) in the cohort representing a total of 13,354 stool samples analyzed between 3 months and 8 years of age. Stool sampling times for detection of STH by microscopy (and numbers of stool samples at each time) were at 3 (1,299), 7 (1,619), 13 (1,951), 18 (1,023), 24 (1,726), 30 (608), 36 (1,562), 60 (1,802), and 96 (1,764) months. A total of 33 hookworm infections were detected by microscopy, and positivity rates for each time point from 3 to 96 months were 0%, 0%, 0.2%, 0.3%, 0.2%, 0.2%, 0.5%, 0.5%, and 0.3%, respectively. Hookworm positivity rates for the random subsample analyzed for STH infections by qPCR using primers specific for *Ancylostoma* spp. and *N. americanus* as described previously^8^ were 0% (0/211) at 7 months, 0% (0/267) at 13 months, 0.5% (2/400) at 24 months, 1.8% (5/273) at 36 months, 1.8% (4/217) at 60 months, and 4.5% (14/313) at 96 months.

**Figure 3.**
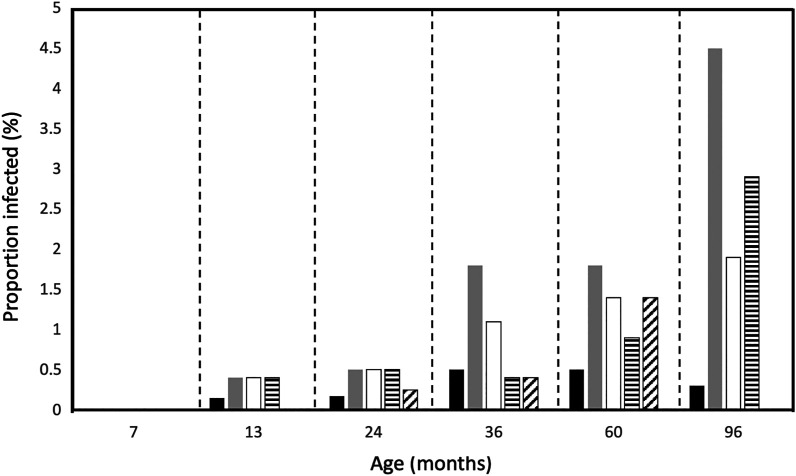
Changes in proportions of children infected with hookworm infection by age in the birth cohort. Shown are proportions of the whole cohort with hookworm using microscopic methods (black bars) and, among the subsample analyzed by qPCR proportions infected with any hookworm species (grey bars), *Ancylostoma ceylanicum* (clear bars), *Ancylostoma duodenale* (horizontal dash bars), and *Necator americanus* (diagonal dash bars). qPCR = quantitative polymerase chain reaction.

Proportions infected with hookworm and individual hookworm species for sampling times from 7 to 96 months are shown in Figure 3. Proportions infected tended to increase with age to 8 years, being much greater with PCR detection than with microscopy. Archived samples were available for analysis of zoonotic and anthropophilic hookworms from 57 of 62 hookworm-positive samples (detected by microscopy in the whole cohort or by previous qPCR in the subsample). Of the five samples not included, one was a duplicate positive between the two analyses and four were missing. No child had *A. ceylanicum* detected on more than one occasion. Two children had hookworm infection detected at two observation times; only one of these was infected with the same hookworm species (*N. americanus*) at both time points. Detailed information on travel and migration histories was available for the children with *A. ceylanicum* infections; apart from internal migrations within the study district (Quinindé, Esmeraldas Province) during 8 years of follow-up, none had spent prolonged periods outside the district, including travel outside Ecuador.

## DISCUSSION

Humans can become infected with anthroponotic (*N. americanus* and *A. duodenale*) and several zoonotic species, including *A. ceylanicum*, *A. braziliense*, and *Ancylostoma caninum*.^19^ *Ancylostoma ceylanicum* is considered the only zoonotic species capable of completing its life cycle in humans.^3^ Although hookworms of humans can be speciated based on morphology, such studies are relatively rare, requiring specific expertise and the availability of adult worms obtained after expulsion or cultivation of L3 larvae from feces. Of the other zoonotic hookworms, *A. caninum* infections are considered to cause cutaneous larva migrans and eosinophilic enteritis^20^ but are thought not to reach egg patency in humans,^21^ and *A. braziliense* causes cutaneous larva migrans.^22^

The application of highly specific molecular detection methods has permitted better understanding of the geographic distributions and epidemiology of different hookworm species in human populations.^16^^,^^19^^,^^23^^–^^25^ All three species of hookworms infecting humans are globally distributed, including *A. ceylanicum*, which has been described in Asia, Oceania, Africa,^2^^,^^26^^,^^27^ and more recently the Americas.^4^^,^^5^

In this retrospective analysis, done in human populations in Ecuador using stool samples collected and archived between 2005 and 2017 from five studies done in eight districts in six provinces from coastal, Andean, and Amazon geoclimatic regions of Ecuador, we detected infections with all three species. Of these, *A. duodenale* and *N. americanus* were found throughout the coastal region and in the Andean and southern Amazon regions, whereas *A. ceylanicum* was found in northern coastal and southern Amazon regions. A previous study detected *A. ceylanicum* in six children living in a marginalized urban setting in the same northern coastal region as in this study.^4^

Although *A. duodenale* was the most frequent hookworm detected (34.9% of all samples tested), among samples positive for hookworm eggs by microscopy the proportions positive for the three hookworm species were similar (i.e., approximately one-third infected with each species). Co-infections with other hookworm species and other STH parasites were frequent among hookworm-infected individuals. Overall, hookworm infections were relatively infrequent in the studies from which these samples were derived (< 4% by microscopy), and data from the birth cohort in a rural district in a tropical coastal region of the country (Quinindé, Esmeraldas Province) showed the infection to be of low endemicity in that specific setting (i.e., < 5% prevalence at 8 years when detected by qPCR); only two children had repeated hookworm infections documented by microscopy during childhood in this cohort of more than 2,000 children, and infections in the other children were detected at only a single time point during the first 8 years of life.

Previous studies in the Latin American region have demonstrated the presence of both *N. americanus* and *A. duodenale*, with a general preponderance of the former: 1) hookworm adults detected on duodenoscopy from a severely anemic patient from a subtropical region of the Andes were identified morphologically as *A. duodenale*^28^; 2) both *N. americanus* and *A. duodenale* (73% versus 15%, respectively, with 12% of infections being mixed) were identified by microscopy of L3 larvae in 192 samples from two rural areas in Itaugua, Paraguay^29^; 3) *N. americanus* (but not *A. duodenale*) was detected by multiplex PCR in 10% of stool samples collected from 228 children and adults living in a rural community from central northern Venezuela^30^; 4) in Argentina, *N. americanus* only was detected by PCR in 32.8% of 140 samples collected from Buenos Aires and Misiones Provinces,^31^ whereas a survey of 99 samples detected both *N. americanus* and *A. duodenale* (36.4% versus 19.1%, respectively, with 48.6% of hookworm infections being mixed infections) in Salta Province^32^; and 5) both *N. americanus* and *A. duodenale* (42.5% versus 7.5%, respectively, but no mixed infections) were detected using semi-nested PCR in 40 samples from an unspecified location in Brazil.^33^ A recent systematic review suggested that worldwide, *N. americanus* is the dominant species (79% of infections versus 32% for *Ancylostoma* spp.), with a similar pattern being present in Latin America.^2^

Hookworm infection rates, particularly with *Ancylostoma* spp., have declined worldwide since the 1990s.^1^ Similarly, infection rates with hookworm have declined dramatically in northwestern Esmeraldas Province, where infection rates in different studies (and study populations) varied between 39%^34^ and 77%^35^ in samples of adults and children up to the 1990s but declined to 5.4% in schoolchildren by 2008.^36^ Such declines likely reflect increasing economic development and improved access to medications and health services over this period. A national survey of schoolchildren done between 2011 and 2012 in coastal, Andean, and Amazon regions estimated a national hookworm infection rate of 5.0% (with 91.1% being low-intensity infections) but hookworm prevalences of 0%, 0.3%, and 14.6% in coastal, Andean, and Amazon regions, respectively.^37^ This compares with a national prevalence rate of hookworm of 33% in a survey of 1,568 stool samples examined in health centers in 1982 (coastal 42%, Andean 0%, and Amazon 35%).^38^ However, all these studies used relatively insensitive microscopic methods and likely underestimated prevalence compared with more sensitive molecular methods,^39^ particularly at low-infection intensities. For example, in the cohort studied here, the estimated prevalence of hookworm in children at 8 years using a combination of microscopic methods was 0.3% compared with 4.5% by qPCR. Furthermore, relatively high rates of hookworm infection (33.1%) were detected by qPCR among samples that were negative for hookworm by microscopy, indicating an important undetected burden of hookworm infection. In Ecuador, the Amazon region not only provides optimal climatic conditions for hookworm transmission, being hot and humid throughout the year, but also is the poorest region of Ecuador.^37^ Lack of potable water, sanitation, and rubbish collection and the presence of dogs indoors and outdoors are typical of Amazonian communities.^40^ It is likely that hookworm remains highly endemic in geographically isolated populations living in extreme poverty in this region, including among indigenous peoples.

This study has some important limitations. Although providing novel data on the presence and geographic distributions of zoonotic and anthropophilic hookworms in Ecuador, this retrospective analysis was restricted by the availability of suitably preserved samples allowing molecular analysis, which likely biased detection of hookworm species to specific localities and population groups favored by researchers. It is highly likely that we have omitted unstudied geographic regions where hookworms are present. We were able to include only a small number of archived samples from a single survey in the Amazon region—known to be the most endemic region nationally^37^—where factors of climate and extreme poverty, particularly among some indigenous peoples, are likely to favor transmission. Without representative sampling from all geographic regions of the country, our data should be interpreted with caution when inferring the relative distributions of species at a national level. Despite such limitations, the study samples did include populations from the three main geoclimatic regions of the country (i.e., coastal, Andean, and Amazon) and populations with varying ethnicities (i.e., Afro-Ecuadorian, indigenous, and mestizo). Stool samples for four of the studies were included based on the availability of archived samples positive for hookworm infection by microscopy, whereas samples from the fifth study (all hookworm negative by microscopy) were included to increase geographic range of sampling. In the latter, we were able to detect and speciate hookworm infections by more sensitive qPCR. Samples had been stored for periods of up to 15 years, and sample quality (either before the addition of preservative or during storage) may have deteriorated, limiting our ability to detect parasite DNA. This might explain why we were able to detect hookworm by qPCR in 82.6% of microscopy-positive samples, although we cannot exclude observer error at microscopy. Detailed data on migration history were available only for the birth cohort; thus, we cannot exclude migration of infected individuals from other regions in the other surveys. A strength of the study was the use of samples from a birth cohort that allowed us to better understand the epidemiology of these hookworm species longitudinally during childhood, although only in one specific setting. However, because stool samples were collected periodically during childhood in the cohort, STH infection prevalence, including with hookworm, may have been reduced by frequent anthelmintic treatments with albendazole for positive stool samples for STH parasites.

These findings extend our previous findings of human *A. ceylanicum* infections in older (i.e., > 20 years) archived samples from a restricted geographic region in northwestern coastal Ecuador (Esmeraldas Province)^4^ to show that *A. ceylanicum* infections are transmitted more widely than previously known both within the same province and in the Amazon region of the country as well. Our observations also have implications for control and elimination strategies for a zoonotic STH infection with domestic animal reservoirs. A combination of factors, including a lack of appropriate veterinary care for dogs (and cats) in these settings and the nature of human–dog interactions accompanied by lack of sanitation, will favor the transmission of *A. ceylanicum*.

In conclusion, our findings show the presence of human infections with *A. ceylanicum* in coastal and Amazon regions, whereas the anthropophilic hookworms appear to be more widely distributed. Over the past 20 or so years, hookworm prevalence and infection intensities likely declined through economic development, improved living conditions, and access to drinking water, sanitation, and health services in many regions of the country. Hookworm prevalence would therefore be expected to have declined since sampling was done in each of the locations included in this analysis. Hookworm infections, however, are likely to remain highly endemic in some settings, particularly in geographically isolated populations living in extreme poverty in the Amazon region. Further studies are required to define the geographic distribution of *A. ceylanicum*, potential risk factors including animal reservoirs, and its public health relevance as a human pathogen in more highly endemic settings in Ecuador and elsewhere in the Americas, where the presence of zoonotic transmission may require additional strategies to those currently being implemented for control of nonzoonotic STH infections.

## Supplemental Materials

10.4269/ajtmh.23-0469 Supplemental Materials
