# Supplementary material for: Geographic Distribution of Human Infections with Zoonotic Ancylostoma ceylanicum and Anthropophilic Hookworms in Ecuador: A Retrospective Analysis of Archived Stool Samples
Source: Am J Trop Med Hyg. 2024 Jan 23;110(3):460–9. doi: 10.4269/ajtmh.23-0469 (PMC10919174; doi:10.4269/ajtmh.23-0469)
Supplement: Supplemental Materials [file tpmd230469.SD1.pdf]

Table S1. Primer and probe sequence information for the six parasites tested.

Parasite	Forward primer sequence, 5'→3'	Reverse primer sequence, 5'→3'	Probe sequence 5'→3'
Ascaris lumbricoides	CTTGTACCACGATAAAGGGCAT	TCCCTTCCAATTGATCATCGAATAA	56-FAM/TCTGTGCAT/ZEN/TATTGCTGCAATTGGGA/3IABkFQ
Ancylostoma ceylanicum	CAAATATTACTGTGCGCATTTAGC	GCGAATATTTAGTGGGTTTACTGG	56-FAM/ CGGTGAAAG/ZEN/CTTTGCGTTATTGCGA/3IABkFQ
Strongyloides stercoralis	CGCTCCAGAATTAGTTCCAGTT	GCAGCTTAGTCGAAAGCATAGA	56-FAM/ACAGTCTCC/ZEN/AGTTCACTCCAGAAGAGT/3IABkFQ
Trichuris trichiura	GGCGTAGAGGAGCGATTT	TACTACCCATCACACATTAGCC	56-FAM/TTTGCGGGC/ZEN/GAGAACGGAAATA TT/3IABkFQ
Necator americanus	CCAGAATCGCCACAAATTGTAT	GGGTTTGAGGCTTATCATAAAGAA	56-FAM/CCCGATTTG/ZEN/AGCTGAATTGTCA AA/3IABkFQ
Ancylostoma duodenale	GTATTTCACTCATATGATCGAGTGTTC	GTTTGAATTTGAGGTATTTCGACCA	56-FAM/TGACAGTGT/ZEN/GTCATACTGTGGA AA/3IABkFQ

Table S2. Primer sequences for amplification of COX1 gene of *Ancylostoma ceylanicum*.

Parasite	Forward primer sequence, 5'→3'	Reverse primer sequence, 5'→3'
Ancylostoma ceylanicum	GCT TTT GGT ATT GTA AGA CAG	CTA ACA ACA TAA TAA GTA TCA TG

Table S3. Socio-demographic characteristics and co-infections with soil-transmitted helminths characteristics among 26 individuals with positive stool samples for *A. ceylanicum.*

Number	Community	District	Age (yrs)	Sex	Ethnicity	Rural	*A.ceylanicum*	*A.duodenale*	*N. americanus*	*S.stercoralis*	*T.trichiura*	*A.lumbricoides*
1	La Union	Quininde	1	Male	Mestizo	Urban	Yes	No	No	No	No	Yes
2	Quininde	Quininde	1	Female	Afro-Ecuadorian	Urban	Yes	Yes	No	No	No	Yes
3	Cupa	Quininde	1	Male	Afro-Ecuadorian	Urban	Yes	No	No	No	No	Yes
4	Malimpia	Quininde	5	Male	Mestizo	Rural	Yes	No	No	No	No	Yes
5	La Union	Quininde	5	Male	Mestizo	Urban	Yes	No	No	No	No	Yes
6	El Capricho	Quininde	5	Female	Mestizo	Rural	Yes	No	No	No	No	Yes
7	La Marujita	Quininde	5	Male	Mestizo	Rural	Yes	No	Yes	No	Yes	Yes
8	Pueblo Nuevo	Quininde	3	Female	Mestizo	Rural	Yes	No	No	No	No	Yes
9	La Union	Quininde	3	Male	Mestizo	Urban	Yes	No	No	No	No	Yes
10	La Concordia	Quininde	8	Male	Afro-Ecuadorian	Urban	Yes	No	No	Yes	No	No
11	La Union	Quininde	8	Male	Mestizo	Urban	Yes	Yes	No	No	No	No
12	El Mirador	Quininde	2	Male	Mestizo	Rural	Yes	No	No	No	No	No
13	Cube	Quininde	6	Female	Afro-Ecuadorian	Rural	Yes	No	Yes	No	Yes	Yes
14	Sabalito	Quininde	2	Female	Mestizo	Rural	Yes	No	No	No	No	Yes
15	El Duana	Quininde	5	Male	Mestizo	Rural	Yes	Yes	No	No	No	Yes
16	Nuevo Azuay	Quininde	2	Male	Mestizo	Rural	Yes	No	No	Yes	No	Yes
17	La Union	Quininde	1	Male	Mestizo	Urban	Yes	Yes	No	No	No	Yes
18	La independencia	Quininde	3	Male	Mestizo	Urban	Yes	Yes	No	No	No	Yes
19	La Cumbre	Quininde	7	Male	Mestizo	Rural	Yes	Yes	No	No	Yes	Yes
20	Quininde	Quininde	5	Male	Mestizo	Urban	Yes	No	No	No	No	Yes
21	La Union	Quininde	5	Male	Mestizo	Urban	Yes	No	No	No	No	Yes
22	Quininde	Quininde	8	Male	Mestizo	Urban	Yes	Yes	No	No	Yes	Yes
23	Nuevo Azuay	Quininde	8	Male	Mestizo	Rural	Yes	No	No	No	No	No
24	Chucaple	Quininde	7	Male	Afro-Ecuadorian	Rural	Yes	No	No	No	Yes	Yes
25	Jempikat	Tiwintza	15	Male	Indigenous/Shuar	Rural	Yes	Yes	Yes	No	Yes	Yes
26	El Progreso	Eloy Alfaro	8	Male	Afro-Ecuadorian	Rural	Yes	Yes	No	No	No	Yes

Table S4. Distance matrix for phylogenetic analysis comparing Cox1 sequences of *Ancylostoma ceylanicum* from 18 subjects with reference sequences.

	7000_N5	7021_N6	7061_N9	7178_N4	7181_N8	7327_N6	7331_N9	7391_N8	7423_N9	7652_N10	7816_N10	7890_N9	8103_N9	8366_N9	8505_N9	8905_N4	9021_N8	B0_29	ON773157.1 Ancylostoma_ceylanicum	DQ438069.1 Ancylostoma_braziliense	MK271367.1 Ancylostoma_duodenale
7000_N5	100%	100%	100%	100%	100%	100%	100%	100%	99%	100%	98%	100%	100%	100%	100%	100%	98%	100%	99%	39%	41%
7021_N6	100%	100%	100%	100%	100%	100%	100%	100%	100%	100%	98%	99%	100%	100%	100%	100%	98%	100%	99%	39%	41%
7061_N9	100%	100%	100%	100%	100%	100%	100%	100%	100%	100%	98%	100%	100%	100%	100%	100%	98%	100%	99%	39%	41%
7178_N4	100%	100%	100%	100%	100%	100%	100%	100%	100%	100%	98%	100%	100%	100%	100%	100%	98%	100%	99%	39%	41%
7181_N8	100%	100%	100%	100%	100%	100%	100%	100%	100%	100%	98%	99%	100%	100%	100%	100%	98%	100%	99%	39%	41%
7327_N6	100%	100%	100%	100%	100%	100%	100%	100%	100%	100%	98%	100%	100%	100%	100%	100%	98%	100%	99%	39%	41%
7331_N9	100%	100%	100%	100%	100%	100%	100%	100%	100%	100%	98%	100%	100%	100%	100%	100%	98%	100%	99%	39%	41%
7391_N8	100%	100%	100%	100%	100%	100%	100%	100%	99%	100%	98%	100%	100%	100%	100%	100%	98%	100%	99%	39%	41%
7423_N9	99%	100%	100%	100%	100%	100%	100%	99%	100%	100%	98%	99%	100%	100%	100%	100%	98%	100%	99%	39%	41%
7652_N10	100%	100%	100%	100%	100%	100%	100%	100%	100%	100%	98%	99%	100%	100%	100%	100%	98%	100%	99%	39%	41%
7816_N10	98%	98%	98%	98%	98%	98%	98%	98%	98%	98%	100%	98%	98%	98%	98%	98%	100%	98%	98%	38%	40%
7890_N9	100%	99%	100%	100%	99%	100%	100%	100%	99%	99%	98%	100%	100%	100%	99%	100%	98%	99%	99%	39%	41%
8103_N9	100%	100%	100%	100%	100%	100%	100%	100%	100%	100%	98%	100%	100%	100%	100%	100%	98%	100%	99%	39%	41%
8366_N9	100%	100%	100%	100%	100%	100%	100%	100%	100%	100%	98%	100%	100%	100%	100%	100%	98%	100%	99%	39%	41%
8505_N9	100%	100%	100%	100%	100%	100%	100%	100%	100%	100%	98%	99%	100%	100%	100%	100%	98%	100%	99%	39%	41%
8905_N4	100%	100%	100%	100%	100%	100%	100%	100%	100%	100%	98%	100%	100%	100%	100%	100%	98%	100%	99%	39%	41%
9021_N8	98%	98%	98%	98%	98%	98%	98%	98%	98%	98%	100%	98%	98%	98%	98%	98%	100%	98%	98%	38%	40%
B0_29	100%	100%	100%	100%	100%	100%	100%	100%	100%	100%	98%	99%	100%	100%	100%	100%	98%	100%	99%	39%	41%
ON773157.1 _Ancylostoma_ceylanicum	99%	99%	99%	99%	99%	99%	99%	99%	99%	99%	98%	99%	99%	99%	99%	99%	98%	99%	100%	38%	41%
DQ438069.1 _Ancylostoma_braziliense	39%	39%	39%	39%	39%	39%	39%	39%	39%	39%	38%	39%	39%	39%	39%	39%	38%	39%	38%	100%	93%
MK271367.1 _Ancylostoma_duodenale	41%	41%	41%	41%	41%	41%	41%	41%	41%	41%	40%	41%	41%	41%	41%	41%	40%	41%	41%	93%	100%
